# FORMAL TRAINING IN TELEHEALTH UNIQUELY PREPARES AN INTERDISCIPLINARY WORKFORCE IN GERIATRICS AND GERONTOLOGY

**DOI:** 10.1093/geroni/igz038.1014

**Published:** 2019-11-08

**Authors:** Kathryn A Nearing, Hillary D Lum, Janna Hardland, Skotti Church, Stephanie Hartz

**Affiliations:** 1 University of Colorado Anschutz Medical Campus, Division of Geriatrics, Aurora, Colorado, United States; 2 Rocky Mountain Regional VA Medical Center, Aurora, Colorado, United States

## Abstract

Geriatric Research Education and Clinical Centers (GRECCs) are centers of excellence funded by the Veterans Administration for the advancement and integration of research, education and clinical activities in geriatrics and gerontology to improve the health, and health care of, older Veterans. The GRECC Connect program expands access to care for rural-residing older Veterans and caregivers. Eastern Colorado GRECC Connect integrates Associated Health Trainees (Audiology, Psychology, Social Work, Pharmacy) and Geriatric Medicine Fellows into interdisciplinary tele-geriatric and tele-palliative care consultations provided to outlying community-based outpatient clinics. The formal telehealth training includes: (1) an initial didactic orientation to introduce skills, common challenges, and important tips when working with older patients and caregivers via telehealth; (2) direct observation and modeling by preceptors, followed by a structured opportunity to debrief what the trainee observed and address questions; and (3) opportunities to provide Geriatric telehealth services, supported by the interprofessional team and feedback and reflection. A formal competency assessment, standardized observation protocol and debriefing guide support the development and assessment of telehealth competencies. During exit interviews, trainees indicated that these experiences offered unique opportunities to develop their clinical skills, particularly related to active listening and communication. They identified their involvement with GRECC Connect as a highly valued aspect of their Geriatrics training. Rigorous training in telehealth is an essential aspect of workforce development in Geriatrics and Gerontology given the concentration of older adults in rural areas. During this session, we will highlight the value of telehealth training for workforce development in Geriatrics and Gerontology.

